# Volumineuse hydrocèle unilatérale avec ulcération scrotale: à propos d’un cas à l'hôpital Régional de Gao

**DOI:** 10.11604/pamj.2018.30.257.16373

**Published:** 2018-08-07

**Authors:** Mahamadou Mallé, Aliou Moussa Coulibaly

**Affiliations:** 1 Service de Radiologie, Hôpital Régional de Gao, Gao, Mali; 2 Service d'Urologie, Hôpital Régional de Gao, Gao, Mali

**Keywords:** Hydrocèle, unilatérale, ulcération, scrotum, échographie, Hydrocele, unilateral, ulceration, scrotum, ultrasound

## Abstract

Hydrocele is an accumulation of fluid between the testis and its vaginal coat. It may be very abundant. It commonly affects people who live in tropical regions. We report the case of a 56-year old patient, Mr. YM, living in the sahelian zone with very abundant hydrocele and thickening of vaginal coat associated with bag ulcer. The patient presented with big bag associated with a feeling of heaviness as well as annoyance when walking, evolving for about one year with progressive increase in volume. Clinical examination showed voluminous left bag (A,B) with a plague measuring 3 cm (A). Two diagnosis were suspected: voluminous hydrocele (more probable) and severe scrotal tumor. Ultrasound of the bag showed very abundant effusion of in the vaginal coat (3000 ml) with thickening of the tunica vaginalis measuring 7 mm in diameter. The diagnosis of very abundant unilateral hydrocele associated with pachyvaginalitis was retained. The extractions of 2.8 L of stained fluid was performed during surgery (C). Postoperative course was favorable (D) under triple antibiotic therapy.

## Image en médecine

L'hydrocèle est une accumulation de liquide entre les deux feuillets de la vaginale testiculaire. Elle peut être de très grande abondance. Elle demeure une pathologie fréquemment retrouvée en région tropicale. Nous rapportons un cas d'hydrocèle de très grande abondance avec épaississement de la vaginale chez un sujet âgé de 56 ans en zone sahélienne avec ulcération de la bourse. Il s'agit de Monsieur YM âgé de 56 ans ayant consulté pour grosse bourse avec une sensation de pesanteur, gênante à la marche, évoluant depuis un an environ avec une augmentation progressive du volume. À l'inspection, on retrouve une volumineuse bourse gauche (A,B) avec une plaie de 3 cm (A) . Nous avions évoqué deux hypothèses diagnostiques, une hydrocèle volumineuse et une tumeur scrotale ulcérée. Une échographie des bourses réalisée a montré un épanchement de grande abondance dans la vaginale estimée à 3000 ml avec un épaississement de la tunique vaginale dont le diamètre mesure 7 mm. Le diagnostic d'hydrocèle unilatérale de très grande abondance fut retenu avec pachy-vaginalite. L'intervention chirurgicale a extrait 2,8 L de liquide teinte (C) avec une suite opératoire favorable (D) sous triple antibiothérapie.

**Figure 1:**
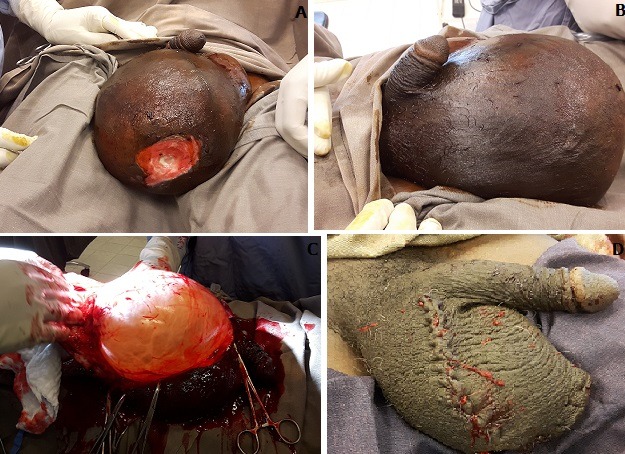
(A) grosse bourse avec ulcération scrotale; (B) grosse bourse vue de profil; (C) liquide teinte en per opératoire; (D) bourse en post opératoire

